# Prediction of Service Performance Based on Physical Strength in Elite Junior Tennis Players

**DOI:** 10.3389/fpsyg.2022.898224

**Published:** 2022-05-20

**Authors:** Nahoko Koya, Tetsu Kitamura, Hiroo Takahashi

**Affiliations:** ^1^Department of Liberal Arts and Sciences, Daido University, Nagoya, Japan; ^2^Faculty of Sports, Biwako Seikei Sport College, Otsu, Japan; ^3^Faculty of Sports and Budo Coaching Studies, National Institute of Fitness and Sports in KANOYA, Kanoya, Japan

**Keywords:** tennis, service speed, physical strength, broad jump, medicine ball throw, multiple regression analysis

## Abstract

In tennis, service requires a variety of complicated movements. Given the importance of taking the initiative to obtain points in a tennis match, it is crucial to make full use of speed and spin rate of service. Generally, a service that requires a higher spin rate would slow down, and a service that has increased speed would have a decreased spin rate. For players who are disadvantaged in height, although controlling spin rate is essential, slowing down service speed should be avoided. For these players, the challenge of service is to improve the speed without decreasing the spin rate. Players must also be trained to build physical strength required for this skill. It is not uncommon to work on physical training without a racket; however, few studies have reported on the effects of cultivated physical strength on on-court tennis performance. Therefore, this study aimed to propose physical measurements that could be used as indices to improve service performance in 58 elite Japanese junior male players. To test service performance, we used TrackMan tennis radar device to assess speed, spin rate, impact height, and impact depth. To test physical strength, we measured 5- and 20-m sprint, broad jump, medicine ball throw (forward, backward). We used a significant multiple regression equation to predict the first service speed obtained from the broad jump and the Medicine ball throw (backward). Additionally, a strong correlation was obtained between the predicted and measured values. In addition to physical strength, we suggest that the depth of the impact point (taking the hitting point forward toward the net) is important for improving the first service speed. However, we were not able to identify the physical strength test items that improve service spin rate. Other item should be examined in the future to determine the physical strength associated with spin rate. This result could help connect physical training and service performance.

## Introduction

The modern game of tennis has evolved from a primarily technical sport to an explosive sport (Ulbricht et al., 2016). Tennis has increasingly become faster and more dynamic, requiring increased strength, speed, and power to achieve higher stroke and serve velocities. The service game has, therefore, become a key factor in game success (Reid and Schneiker, 2008; Gillet et al., 2009; Kovacs and Ellenbecker, 2011). In particular, service is the only stroke in tennis that is entirely under the player’s control and is the most powerful and important shot. After the toss-up, while moving the power from the lower half of the body to the upper half using the whole-body movement chain, the racket speed is increased toward impact (Kibler, 2009). This racket motion can create a mix of ball speed and spin, and a clear trade-off between these has been reported (Sakurai et al., 2007, 2013). Indeed, serving speed is decreased when spin is applied, and vice versa. A high correlation has been reported between the service speed and percentage of points won. According to Fett et al. (2017) and Kramer et al. (2017), the maximal service speed is the most appropriate on-court predictor of player performance. Service reportedly affects the overall game results for male and female players, and service speed is highly correlated with an athlete’s competition level (Whiteside et al., 2013; Ulbricht et al., 2016). This is because an increasing serve speed reduces the time for the receiver to hit the return precisely, and it is possible to take advantage of the following hits and get a direct point (O’Donoghue and Brown, 2008; Vaverka and Cernosek, 2013; Whiteside and Reid, 2017).

To achieve higher speeds during service, the player must account for the net height, position of the service line, and height of the impact point (Koya et al., 2021). Brody (1987) reported that a minimum height of 2.74 m on the baseline is required to eliminate gravity and ball aerodynamics concerns. To get a higher impact point, a player needs to be anthropometrically tall or jump higher, as there is a positive correlation between the impact height and service speed (Girard et al., 2005; Vaverka and Cernosek, 2013; Bonato et al., 2015; Dossena et al., 2018; Hayes et al., 2018). Tall players have the advantage of being able to hit the service at a higher height and in a wider service area (Vaverka and Cernosek, 2013). They are able to serve with an emphasis on ball speed rather than ball spin, without worrying about the net. In addition, taller players have longer upper limbs that enables them to achieve increased speeds with a kinetic chain, using a stronger moment arm (Bahamonde, 2000). However, players with shorter height are required to compensate to impact height by improving their jumping power. Moreover, players are required to improve their spin rate to compensate for insufficient height. Spinning is therefore particularly essential for shorter athletes; however, speed is also required to some extent. As of July 2020, the average height of the top 50 ranking players in the International Tennis Federation was 188.73 cm (International Tennis Federation, 2021), whereas that of the four Japanese players in the top 100 was 176.50 cm (International Tennis Federation, 2021). Japanese players, who are generally shorter, must therefore acquire this technical skill associated with service performance to reach global elite status (Koya et al., 2021). However, it has also been reported that the world’s top players can serve at a high speed while maintaining a high spin rate (Muramatsu et al., 2010, 2015). Thus, it is important for Japanese athletes to learn to improve both the spin rate and speed of their service, and it is necessary to train the physical strength systematically of these athletes in order to make this possible.

Technical skills are predominant factors in tennis (Groppel, 1992; Smekal et al., 2001); thus, players spend much time on the court for technical training with a racket; however, it is difficult to devote the same time to on-court technical and physical training without a racket. However, as the level of competition increases, many players realise the importance of physical strength and fitness (Smekal et al., 2001; Reid and Schneiker, 2008; Fernandez-Fernandez et al., 2009, 2018). Functional links observed between muscular strength in the dominant upper and lower limbs and ranking position in competitive tennis players reinforce the notion that physical characteristics have a strong influence on tennis performance and may be important determinants for successful participation in elite tennis (Girard and Millet, 2009; Fernandez-Fernandez et al., 2013; Ulbricht et al., 2016). The overhead medicine ball throw (MBT), for example, which tests upper-body power as a factor that influences service speed, has been widely used (Kramer et al., 2017; Colomar et al., 2020; Fett et al., 2020). MBT is comparable to training in tennis because this movement requires coordination of energy transfer using a kinetic chain. However, since the kinetic chain of the service in tennis is not the same as that in an MBT perfectly, studies have reported different results depending on the age and level of the players. A study that proposed that an original MBT shot put is similar to serving using a kinetic chain, targeting male professional players, reported that 86% of the service speed could be explained by the MBT result (Sánchez-Pay et al., 2021). In the case of male players, there seems to be no doubt about the relationship between MBT and service speed (Kramer et al., 2017; Colomar et al., 2020; Fett et al., 2020). However, it has not been scientifically proven that the service speed could be predicted by the distance at which the medicine ball (MB) is thrown when junior players are undergoing systematic physical training. It is important to obtain an index that connects the technical achievement of players to physical training, which may lead to further improvements in training efficiency.

Therefore, this study aimed to present physical strength items that could be used as an index for predicting service performance in tennis, which affects the competitiveness of top Japanese junior players in each age category. It is important to connect the technical achievements of players with physical training to obtain indicators, and further improvements in training efficiency are expected.

## Materials and Methods

### Participants

Fifty-eight male elite tennis players (mean ± standard deviation age, 14.66 ± 1.98 years; height, 169.94 ± 7.17 cm; weight, 60.37 ± 11.13 kg) participated in this study, including top-level junior Japanese players in each generation category. Players were selected by the National Federation’s coaching staff based on their competitive performance, and all players had at least 7 years of tennis training. Under our Institutional Review Board’s policies for the use of human participants in research (Approval No. 199) in accordance with the Declaration of Helsinki, the investigator informed all participants about the benefits and possible risks associated with participation in the study. All participants (or guardians) signed a written informed consent document indicating their voluntary participation. Additionally, self-reported medical histories were obtained from all participants. We examined the history of injuries and determined that it had no effect on this study.

### Design and Procedures

We aimed to examine whether a relationship between service performance (speed, spin, impact height, and impact depth), anthropometry (participant height and weight), and physical conditions (sprint, jump and medicine ball throw) exists. Variables were categorised as service speed, service spin, impact height, impact depth, and physical strength. Five independent variables for physical strength were converted into a principal component analysis (PCA) score, and the correlation with service performance (speed, spin, impact height and impact depth) was analysed.

### Service Performance Test

**FIGURE 1:**
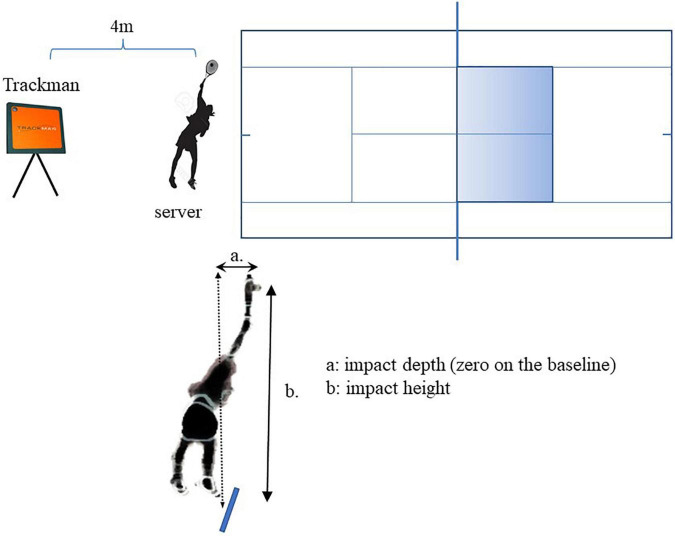
Experimental set up for service performance test and service impact items (Koya et al., 2021).

The service tests were conducted using a TrackMan tennis radar device (TrackMan Inc., Vedbæk, Denmark). This device is an advanced radar that utilises the Doppler effect to capture the behaviour of an object. In this case, the full three-dimensional flight of a tennis ball (speed, spin, spin axis) was recorded. The Doppler effect is an effect in which the relative velocity between a source and an observer results in the shifting of sound or radio wave frequencies (Murata and Takahashi, 2021). If a ball in flight reflects a radio wave, the frequency of the reflected wave shifts, depending on the velocity and spin of the ball, and is calculated using the dedicated software (Martin, 2012). Doppler effect-based measurement devices are superior to other devices in terms of immediacy and user-friendliness and are often used in golf coaching and baseball (Murata and Takahashi, 2021). The accuracy of the TrackMan system is equivalent to that of conventional high-speed cameras or speed radar devices (Martin, 2012; Murakami et al., 2016; Sato et al., 2017; Murata and Takahashi, 2021). This allows for real-time measurements and analysis of parameters, such as spin rate, speed, direction, impact location (height and depth), net clearance, and landing position. In this study, the TrackMan was set at approximately 4 m behind the centre mark, which allowed the radar to visualise the service box (Figure 1).

Service impact was defined as the moment when the ball hit the racket, and the height and depth of the impact point at that moment were measured. The impact depth was analysed with the baseline set to 0 with the forward direction closer to the net considered as positive (+) and backward direction away from the net as negative (–) (Figure 1). The tests were conducted in an indoor tennis court with no returner in place. After a brief warm-up consisting of serves with increasing velocities, players were instructed to perform the first service as fast as possible and the second service with as much spin as possible. Both were directed at the T of the service box. Participants performed three sets of service tests, hitting the first and second serve from the deuce side and then hitting the third serve from the advantage side. The details of the 12 serves per player were collected. For analysis, the average of the six serves was used for each variable. Serves such as faults and nets were excluded, and only successful serves were used.

### Physical Strength Test

Based on the published literature, five variables (5-m sprint, 20-m sprint, broad jump, MBT overhead backward, and MBT overhead forward) related to service performance were measured (Signorile et al., 2005; Kramer et al., 2017; Koya et al., 2018; Delgado-García et al., 2019). In addition to height and weight, the following physical condition variables were measured using the Japan Tennis Association protocols (Koya et al., 2014, 2015):

1. 5- and 20-m sprint: Players were instructed to sprint in a straight line for 20 m; the durations from the starting line to the 5- and 20-m marks were measured using a photocell (Timing Systems by Brower Timing Systems, Draper, UT, United States). In the analysis, the speed was calculated (m/s) based on time. The best values were used.
2. Broad jump: Players performed a jump as far as possible in an open stance with arm action.
3. Medicine ball throw (MBT): Players performed forward and backward (overhead) throws in an open stance with both legs fixed while throwing a 2 kg medicine ball. The best values were obtained.

Test reliability was confirmed by performing a retest (ICC; 5 m sprint:0.75, 20 m sprint:0.91, Broad jump: 0.95, MBT overhead backward: 0.93, MBT overhead forward: 0.97).

### Analyses

Pearson product-moment correlations were conducted to assess the association between the service performance variables (speed, spin rate, impact height, and impact depth) and anthropometric variables (physique and physical strength) (Table 1). We performed PCA using the physical strength test variables and extracted the first principal component score as a comprehensive index of physical general strength (strength PCA) to comprehensively evaluate each player’s physical strength. Table 2 shows the factor loadings, which are the loads of each variable to the strength PCA (Table 2).

**TABLE 1:** The correlation coefficient of physique and physical strength versus service performance.

		1st service					2st service		
	Measured item	speed (km/h)	spin (rpm)	impact H	impact D	speed (km/h)	spin (rpm)	impact H^†^	impact D^†^
physique	Height (cm)	0.53**	−0.20	0.79**	0.28*	0.57**	0.13	0.81**	0.06
	Weight(kg)	0.67**	−0.06	0.56**	0.26	0.45**	0.50**	0.52**	0.03
Strength	5 m sprint (m/sec)	0.47**	0.03	0.22	0.24	0.44**	0.12	0.21	0.12
	20 m sprint (m/sec)	0.72**	0.10	0.37**	0.44**	0.64**	0.24	0.40**	0.29*
	Broad Jump (cm)	0.79**	0.05	0.47**	0.43**	0.64**	0.34**	0.52**	0.16
	MBT overhead backward (m)	0.81**	0.04	0.59**	0.44**	0.72**	0.33*	0.61**	0.26
	MBT overhead forward (m)	0.74**	−0.06	0.52**	0.35**	0.59**	0.36**	0.51**	0.14

r, correlation coefficient; bold notation *p < 0.05, **p < 0.01, ^†^MBT, medicine ball throw; H, height; D, depth.

**TABLE 2:** Factor loading in strength PCA.

	Factor loading in strength PCA	
Strength PCA	5 m sprint (m/sec)	0.682
	20 m sprint (m/sec)	0.908
	Broad jump (m)	0.884
	MBT^†^ overhead backward (m)	0.887
	MBT^†^ overhead forward (m)	0.829

*^†^MBT, medicine ball throw.*

Multiple regression analysis with service performance (speed and spin rate) as the dependent variable and each physical strength test item as the independent variable was performed to determine the physical strength items improving service performance. Based on this, a regression formula was derived for predicting the first service speed. Further multiple regression analysis with strength PCA, impact height, and impact depth as independent variables was performed to determine a regression formula developing the first service speed. No multicollinearity problems were confirmed. Statistical analyses were performed using IBM SPSS Statistics for Windows, version 27 (IBM Corp., Armonk, NY, United States). Statistical significance was set at *p* < 0.05.

## Results

Table 3 shows the results of the service performance and physical strength tests. Regarding the service speed, a correlation between physique and physical strength in the first and second service was found (Table 1). Conversely, the service spin rate in the second service was correlated with physique, jump, and MBT. However, the correlation coefficient was not as high as the speed. No correlation was found between physique and physical strength in the first service spin. Regarding the impact point in the first service, both the impact height and depth were correlated with physique and physical strength; in the second service, only the impact height was correlated with physique and physical strength, whereas the impact depth was only correlated with the 20-m sprint.

**TABLE 3:** Physique, strength test and service performance test results of players.

	Physique					Strength		
	Age (years)	Height (cm)	Weight (kg)	5 m sprint (m/sec)	20 m sprint (m/sec)	Broad jump (m)	MBT overhead backward (m)	MBT overhead forward (m)
	14.66 ± 1.98	169.94 ± 7.17	60.37 ± 11.13	4.47 ± 0.26	6.08 ± 0.28	2.23 ± 0.20	11.53 ± 2.36	8.25 ± 1.80
	
Male players (58)	**Service**							
	
	**1st service speed (km/h)**	**1st service spin (rpm)**	**1st service impact H^†^ (m)**	**1st service impact D^†^ (m)**	**2nd service speed (km/h)**	**2nd service spin (rpm)**	**2nd service impact H^†^ (m)**	**2nd service impact D^†^ (m)**
	
	170.22 ± 15.18	1378.89 ± 334.83	2.60 ± 0.12	0.01 ± 0.18	130.32 ± 13.46	3487.58 ± 518.22	2.56 ± 0.14	−0.26 ± 0.20

*^†^MBT, medicine ball throw; H, height; D, depth.*

**FIGURE 2:**
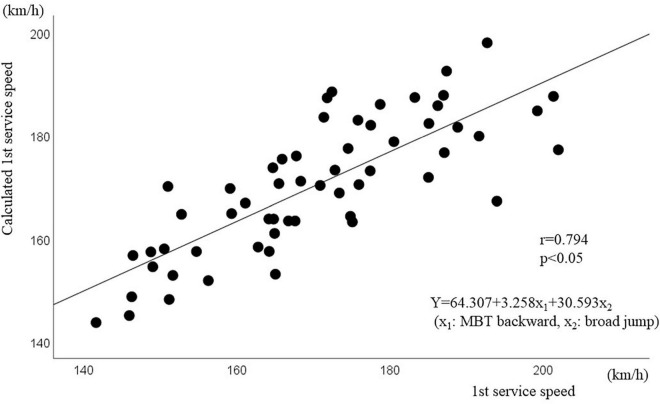
Correlation analysis between the measured first service speed and calculated service speed Estimated formula from MBT backward and broad jump.

Multiple regression analysis was performed to predict the first service speed based on these physical strength test items. Multiple stepwise regression analysis was performed on 58 male tennis players, and multicollinearity was observed. As a result, a significant regression equation [*Y* = 64.307 + 3.258x_1_ + 30.593x_2_ (x_1_: MBT backward, x_2_: broad jump), *F* = 72.298, *p* = 0.001] was found using broad jump and MBT (backward) as independent variables, which could predict the first service speed. The contribution rate of this equation was 72.0% (*r* = 0.851); in other words, 72% of the first service speed could be explained by MBT (backward) and broad jump. Figure 2 shows the estimated value based on this regression equation and the measured value of the first service speed. A strong correlation was found between the estimated and measured values (*r* = 0.794, *p* = 0.001), and the average value of the difference between these two values was 6.38 ± 4.71 km/h.

**FIGURE 3:**
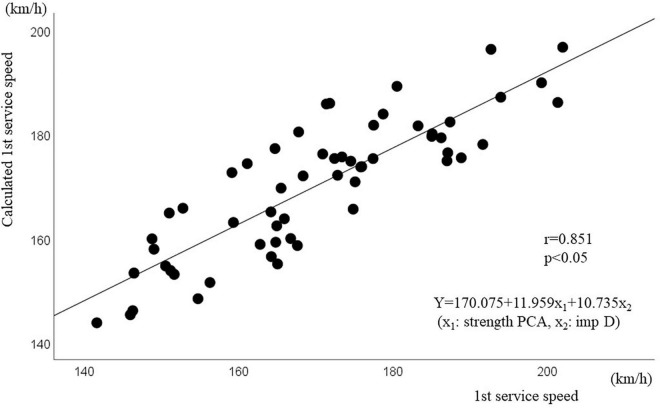
Correlation analysis between the measured first service speed and calculated service speed Estimated formula from strength PCA and impact.

As a comprehensive physical index from the physical strength test, strength PCA was calculated and used for analysis as an independent variable. Impact height and impact depth were set as independent variables, as indices of service skill. Multiple stepwise regression analysis was performed, and multicollinearity was found. As a result, a significant regression equation [*Y* = 170.075 + 11.959x_1_ + 10.735x_2_ (x_1_: strength PCA, x_2_: impact depth), *F* = 73.703, *p* = 0.001] was found with strength PCA and impact D as independent variables, which could also predict the first service speed. The contribution rate of this equation was 72.8% (*r* = 0.853); in other words, 73% of the first service speed could be explained by strength PCA and impact depth. Figure 3 shows the estimated value based on this regression equation and the measured value of the first service speed. A strong correlation was found between the estimated and measured values (*r* = 0.851, *p* < 0.001), and the average value of the difference between these two values was 7.72 ± 6.04 km/h.

## Discussion

### Physical Strength Factors Leading to Service Speed

In this study, a multiple regression formula was obtained for predicting the first service speed using broad jump and MBT (backward) values. Indeed, a strong correlation was shown between the calculated and measured values. Additionally, impact depth (taking the hitting point forward from the baseline) was found to be as important as physical strength to improve the first service speed. Compared to the broad jump and MBT (backward), a similar movement common to service is to use the flexion and extension of the hip joint to exert power. Utilising this flexion and extension of the hip joint, a leg drive is thought to increase the service speed. The tennis service motion has three distinct phases: preparation, acceleration, and follow-through (Kovacs and Ellenbecker, 2011). The acceleration phase, which starts with preparation for power loading to hitting the ball, involves physical factors; this phase marks the beginning of physical strength utilisation for service speed. When the server initiates knee flexion for power loading, a natural stretch-shortening cycle and a combination of eccentric and concentric contractions of the leg muscles help store elastic energy (Girard et al., 2005). This elastic energy can assist the leg drive (Elliott et al., 2009). Indeed, service speed is correlated with a forceful leg drive created by greater muscle forces (Bahamonde, 1997). Elite players can coordinate bilateral extension at the lower limb joints to propel their bodies off the ground during the leg drive (Bahamonde, 2000; Knudson et al., 2004; Girard et al., 2005; Reid and Schneiker, 2008). This lower limb motion contributes significantly to the force required for a tennis serve (Kibler, 2009). The pushing action, which uses a backward-to-forward sequence with higher horizontal forces, as seen in elite-level servers, may be of greatest importance in generating high-speed serves (Girard et al., 2005). A server that effectively uses a leg drive could have a maximum swing speed of 7.4 m/s (Elliott et al., 1986). An effective leg drive could not only increase service speed but also make it possible to hit at a higher impact point to pass over the net at higher point (Elliott et al., 2009). Whiteside et al. also reported that the lesser the leg drive that produces angular momentum connecting to the trunk, the lesser power transmitted to the serve is (Whiteside et al., 2015). In this manner, a previous study clarified that leg drive could be highly related to service speed (Elliott et al., 1986, 2009; Girard et al., 2005; Whiteside et al., 2015); the regression formula in this study is in line with these results.

Previous studies have shown diversity results on the contribution of jumps to service speed (Sebolt, 1970; Bahamonde, 2000; Chow et al., 2003; Cardoso Marques, 2005; Girard et al., 2005; Ferrauti and Bastiaens, 2007; Fett et al., 2020); however, unifying the jump directions could lead to similar results. In this study, the jumps were performed horizontally rather than vertically, which might have greatly contributed to service speed owing to their relationship with leg drive. Sánchez-Pay et al. (2021) reported that although there was a correlation between service speed and countermovement jump, there was no correlation between service velocity and jump height when serving. Moreover, flight time is more important than the jump height (Sánchez-Pay et al., 2021), which might be because the jump is not vertical but horizontal (forward to the net) while serving, in which the impact depth is forward. Considering this mechanism, it is expected that the results of this study showed that service speed is highly correlated with broad jump and impact depth.

As conditions for impact related to service speed, as shown in this study, taking the impact point much higher and more forward is required (Girard et al., 2005). Typically, a tall player has longer upper limbs and the length of the moment arm makes it possible to increase the racket swing speed. In addition, it is possible to take the impact point forward to the net. Conversely, players who are shorter must make up for this by physical training to take the impact point much higher and more forward (e.g., improve the strength of jumping higher and forward). The service form must transit power to the ball *via* the lower limbs, trunk, upper limbs, and racket while efficiently using the kinetic chain of the whole body. MBT has been widely used to train this kinetic chain. MBT is a test item for the power of the upper body, and it also uses the kinetic chain from the lower body to transfer energy to the upper limbs, thus training the entire body for the strength required for serving. While there are some studies on the relationship between service speed and MBT (Kramer et al., 2017; Colomar et al., 2020; Fett et al., 2020), the contribution of MBT to service speed is varied. Comparing the body movement during service, the racket operation is performed using one arm at the end, whereas in MBT, the MB is thrown using both arms. In addition, because the players were not accustomed to the weight of a 2 kg MB and MBT movements were complicated, the results were considered to vary depending on the age, competition level, and sex of the participants. In this study, junior players performed two types of MBT, forward and backward, and MBT in the backward direction was found to predict service speed. Abdominal muscle strength is important for overhead forward throwing, while back muscle strength is required for overhead backward throwing. Since impact depth affects the service speed, stabilizing the trunk could play an important role in the service movement to hit the forward impact point. The results of this study suggested that stabilizing the trunk might lead to a successful overhead swing-like service. A similar movement is required for javelin throwers and baseball pitchers and stability training around the scapula is necessary to rotate the shoulder efficiently (Wilk et al., 1993; Ali and El-Ghaffar, 2010; Kim et al., 2014; Hurd et al., 2017). However, even if some muscle strength is gained, it may not immediately be reflected in the service speed, and it is necessary to efficiently train to transmit power while considering the overall movement balance. When training physical strength without a racket, it is difficult to imagine how the target physical factor affects the shot on the court. It is not easy to imagine how far to jump, how far to throw the medicine ball, and the speed with which this corresponds. As movements of broad jump and MBT are different from service, it is necessary for players to be aware of the kinetic chain of the whole body and to acquire efficient power transmission. Considering this, training and measurement items are desirable so that the value reflect correct and efficient use of the body.

The multiple regression formula shown in this study can predict the first service speed by applying the value of their broad jump and MBT (backward). In order to hit the service of 200 km/h, players could have a standard by which they have to jump and throw in order to aim for during physical training without a racket. An index that connects physical training and on-court performance might make it possible to allow for training that is intimately linked to improving competitiveness. In reality, the strong correlation between the first service speed and broad jump and MBT (backward) does not necessarily guarantee an improvement in service speed. However, if physical training aims to refine the movement of tennis and improve the physical strength required for the technique, such a task setting might be useful in the training field. In addition, as a practical use of this regression formula, it will be possible to prioritize the training program order of players. For example, if the player is plotted on the left side of the regression line, it could be determined that the service should be able to hit at a faster speed, considering their physical strength. In this case, technical improvement is a priority task for these players. In this way, it is thought that it will lead to the formulation of an efficient training plan by determining the priority of whether to train physical strength or improve skills in improving the competitiveness of players. In future studies, a regression formula for each age category of players, such as U12, U14, U16, and U18, could be developed by including more participants. Furthermore, even in racket sports, such as paddle-tennis, a connection can be noted between physical training and skill practice by obtaining a regression formula from the physical strength index and performance index.

### Reasons a Relationship Between Service Spin Rate and Physical Strength Were Not Found

In this study, it was not possible to extract the physical strength factors affecting the spin rate of the service. According to previous study, no correlation was found between service spin and physical strength in male players, but a correlation was reported in female players (Koya et al., 2021). However, due to the small number of participants, further research was needed (Koya et al., 2021). We examined more than 50 players this time, but the results were the same as that in the previous study. The variables of physical strength dealt with in this study were selected based on running, jumping, and throwing, which are the basic movements of sports. In racket sports, it is thought that the swing speed affects the spin rate; however, these test variables, using the movement of the shoulder joint on the dominant side that directly affects swing speed, were not adopted in this study. If there was a variable to measure the physical strength related to the speed of the swinging arm, the relationship between spin rate and physical strength may have been found. In a previous study, female players showed a correlation between spin rate and physical strength; in the future, this should be examined using the additional measurement of racket swing speed. Tall players generally have long limbs. Increased forearm angular momentum improves the forward linear speed of the wrist, accelerating the racket speed, which plays an important role in improving the speed of the ball (Bahamonde, 2000). The forearm of a tennis player is interpreted as a moment arm with the axis of rotation at the elbow, and if this moment arm becomes longer, the tangential speed may increase (Elliott, 1988; Cross, 2004). However, in this study, no significant correlation was found between height and the spin rate. In the future, it is necessary to reconsider the measurement items. This is a limitation of this study. Further investigation using additional measurements of the racket swing speed that directly affect spin rate is needed.

## Conclusion

The present study found that a significant multiple regression equation for predicting the first service speed was obtained from the broad jump and the MBT (backward), and a strong correlation was found between the predicted and measured values. In addition to the physical strength factor, it was suggested that the depth of the impact point (taking the hitting point forward toward the net) is important for improving the first service speed.

## Data Availability Statement

The datasets presented in this article are not readily available because they can only be used with the permission of the Japan Tennis Association National Team. Requests to access the datasets should be directed to NK, n-koya@daido-it.ac.jp.

## Ethics Statement

The studies involving human participants were reviewed and approved by Daido University Ethics Committee. Written informed consent to participate in this study was provided by the participants’ legal guardian/next of kin.

## Author Contributions

NK: conceptualisation, methodology, data collection, formal analysis, writing – original draft preparation, review, and editing. TK and HT: data collection and advising. All authors contributed to the article and approved the submitted version.

## Conflict of Interest

The authors declare that the research was conducted in the absence of any commercial or financial relationships that could be construed as a potential conflict of interest.

## Publisher’s Note

All claims expressed in this article are solely those of the authors and do not necessarily represent those of their affiliated organizations, or those of the publisher, the editors and the reviewers. Any product that may be evaluated in this article, or claim that may be made by its manufacturer, is not guaranteed or endorsed by the publisher.
